# Feasibility to infer accurate fibrinogen concentrations from thrombin generation, prothrombin time, or Reptilase time waveforms

**DOI:** 10.1016/j.rpth.2025.103269

**Published:** 2025-11-19

**Authors:** Ryanne A. Arisz, Bart Bakker, René van den Ham, Aamer Ahmed, Jeroen C.J. Eikenboom, Saskia E.M. Schols, Moniek P.M. de Maat, SYMPHONY consortium@SYMPHONY_NL

**Affiliations:** 1 Department of Hematology, Cardiovascular Institute, Erasmus MC, University Medical Center Rotterdam, Rotterdam, the Netherlands; 2 HEMEO B.V., Amsterdam, the Netherlands; 3 Division of Thrombosis and Hemostasis, Department of Internal Medicine, Leiden University Medical Center, Leiden, the Netherlands; 4 Department of Hematology, Radboud University Medical Center, Nijmegen, the Netherlands and Hemophilia Treatment Center Nijmegen-Eindhoven-Maastricht, Nijmegen, the Netherlands

**Keywords:** fibrinogen, fibrinogen assays, prothrombin time, Reptilase time, thrombin generation

## Abstract

**Background:**

A rapid point-of-care test to measure fibrinogen levels is crucial to adequately treat major blood loss, eg, because of trauma, postpartum hemorrhage, or major surgery. Obtaining results from current fibrinogen assays is too time-consuming in emergency situations. We have developed mathematical methods to infer fibrinogen concentrations from the turbidity waveforms of the thrombin generation (TG), prothrombin time (PT), and Reptilase time (RT) assays.

**Objectives:**

The aim of this study was to verify the performance of these methods using assay waveforms from real-world patient samples.

**Methods:**

We measured TG, PT, and RT in 44 plasma samples with a wide range of fibrinogen levels (1.1-16.6 g/L), high D-dimer, prolonged PT, and/or containing anticoagulant medication.

**Results:**

Overall, data from the PT (R = 0.965) and RT (R = 0.985) assays could be used as data input to accurately infer fibrinogen levels for all patients. The TG assay (R = 0.917) was less robust and generated results for only 36 patients.

**Conclusion:**

Our mathematical methods proved to be able to accurately infer a wide range of fibrinogen levels in real-world patient samples, even in the presence of anticoagulant medication. With this study, we took the first step toward the development of a new, rapid point-of-care fibrinogen test.

## Essentials

• In case of major blood loss, a fast method to measure fibrinogen levels is required.
• Mathematical models can infer fibrinogen levels in blood based on data from clotting assays.
• Data from the PT and RT assays proved to give the most accurate results.
• With this data, our model could accurately infer fibrinogen levels in (challenging) patients.

## 1 Introduction

Fibrinogen is an essential protein for hemostasis. The final step of secondary hemostasis is the conversion of fibrinogen to fibrin, which then forms long fibers and a fibrin network that strengthens the platelet plug and stops the bleeding [1]. Normal plasma levels of fibrinogen range between 2.0 and 4.0 g/L. In case of major blood loss, eg, due to trauma, postpartum hemorrhage, or major surgery, fibrinogen levels can rapidly decrease to critical levels (summarized in Levy et al. [2]). When major bleeding occurs or when there is a high risk of bleeding, guidelines recommend the supplementation of fibrinogen to minimize continuous bleeding or the risk thereof [3]. Therefore, close monitoring of fibrinogen levels in patients with major blood loss is key to adequate treatment.

Today, multiple fibrinogen assays are available. Of these, the Clauss assay is most used. A high concentration of thrombin is used to initiate clotting in this assay [4]. During the clotting process, the time until clot formation is measured either by turbidity or the mechanical resistance of the plasma sample. Clotting times are then compared with those of reference plasma samples with known fibrinogen levels to calculate the fibrinogen concentration in the sample. Due to the high thrombin concentration, the coagulation cascade does not significantly influence the clotting rate. This makes the Clauss assay insensitive to reduced levels of clotting factors and anticoagulant medications such as heparin. As such, a wide variety of patient samples can be measured with the Clauss assay.

Another method used in some laboratories to determine fibrinogen levels in patient plasma is the prothrombin time (PT)-derived assay. The endpoint of this method is the maximum turbidity within a measuring time of 180 seconds of a standard PT assay. Hence, when PT needs to be determined, the fibrinogen concentration for that patient can be obtained at no additional costs or labor. The accuracy and reliability of the assay have been questioned, however. In general, PT-derived fibrinogen levels seem to be in good agreement with the fibrinogen levels measured using the Clauss method, as long as the concentrations are within the normal range and without dysfibrinogenemia [5], depending on the reagent used [[5], [6], [7]]. Another drawback of this assay is that it is inaccurate when PT is prolonged (eg, due to anticoagulant treatment). Therefore, it is not recommended to use PT to derive fibrinogen concentrations, especially if fibrinogen levels are < 2.0 g/L [6].

Many hospitals now use viscoelastic measurements to obtain indicative fibrinogen levels in critical care situations [3]. Results are available within 5 to 10 minutes after initiation of clotting when using this type of measurement, but they are semiquantitative, as the assay is not calibrated for this purpose. As clotting is activated using tissue factor in this assay, fibrinogen concentrations can only be reliably estimated if all other coagulation factor concentrations, besides fibrinogen, are within normal ranges. Despite this concern, previous studies showed that the estimated fibrinogen concentrations of the FIBTEM closely correspond to those of the Clauss assay in multiple distinct study populations [8,9].

In the Reptilase time (RT) assay, fibrinogen is converted to fibrin by batroxobin, which only cleaves fibrinopeptide A and leaves fibrinopeptide B uncleaved. This assay is often used to detect fibrinogen abnormalities or dysfibrinogenemias [10]. Because batroxobin cleaves fibrinogen directly, other coagulation factors and heparin do not affect the RT assay result [10,11].

**Figure 1:**
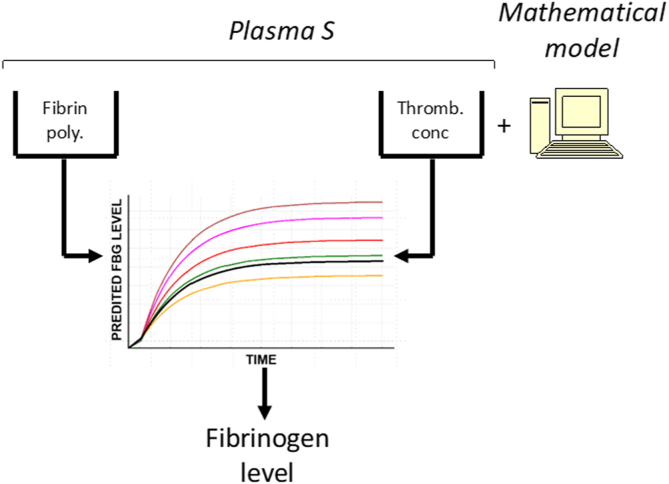
Schematic representation of the thrombin (Tromb.) generation-based method. Fibrin polymerization (poly.) and Thromb. concentration (conc) are simultaneously measured in the same sample (Plasma S). The thrombin conc is used to simulate fibrin poly. using the mathematical model. The fibrinogen (FBG) level of plasma S is then inferred by “fitting” the simulated curves (colored lines) to the measured turbidity curve (black curve).

**Figure 2:**
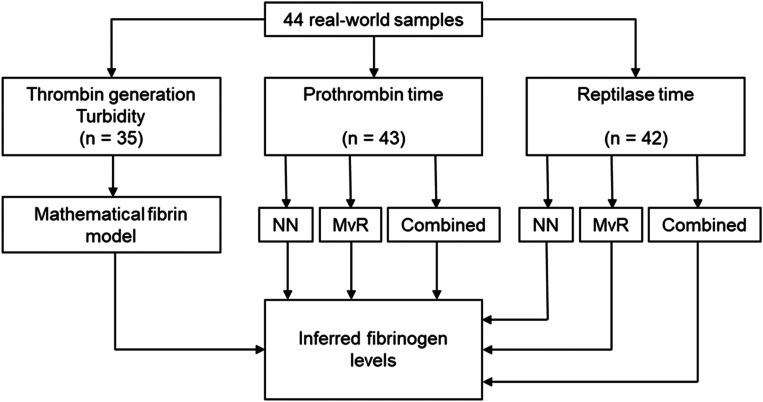
Overview of the different methods that were used to infer the fibrinogen levels in the patient samples. MvR, multivariate linear regression model; NN, neural network model.

To develop a new rapid method to determine fibrinogen levels in plasma, we initially verified a mathematical model to infer the fibrinogen concentration from measurements of increasing turbidity and the developing concentration of activated thrombin upon initiation of the coagulation process in commercially obtained plasma samples [12]. The mathematical model simulates the increase in turbidity of plasma samples by the fibrin polymerizationprocess based on the measured thrombin concentration. It is used to generate a reference curve that describes the relationship between the simulated maximum increase in light attenuation for a series of fibrinogen concentrations. The unknown fibrinogen level in the sample is estimated by projection of the measured maximum turbidity increase in the turbidity assay to the reference curve. A schematic representation of the proposed solution is shown in Figure 1. As this method does not need calibration plasma to determine the fibrinogen level in the sample, it is likely that this method is suitable for further development into a point-of-care (POC) device. In addition, we inferred fibrinogen concentration in plasma samples from PT and RT assays using linear regression and a 3-layer neural network (NN) analysis of their turbidity curves [13]. An overview of the data input and all the methods that were investigated in this stuyd are summarized in Figure 2.

In this study, we will further verify these methods using clinical samples collected for diagnostic testing at the hemostasis laboratory of Erasmus MC. We compared the fibrinogen levels derived from the models with those measured by the Clauss assay. To test the robustness of the method, standard samples with a wide range of fibrinogen levels were used, as were samples from patients receiving anticoagulant treatment, a high D-dimer, and/or prolonged PT.

## 2 Methods

### 2.1 Selection of patients

We collected residual plasma from 44 selected patient samples that were send in for routine diagnostic testing to the hemostasis laboratory at Erasmus MC between September 2021 and October 2021. The selection was based on routine testing for fibrinogen levels, anti-Xa levels, international normalized ratio (INR), D-dimer, or coagulation tests during direct oral anticoagulant (DOAC) use. Patients aged <18 years were not selected. We selected samples with a wide variety of fibrinogen concentrations, abnormal D-dimer or anti-Xa levels, an INR between 2 and 3, or from patients taking any form of DOAC medication at the time of inclusion. After inclusion, diagnostic test results on fibrinogen levels, anti-Xa levels, INR, and D-dimer were collected from electronic patient records, if available, as well as status updates on any medication use that potentially interferes with the coagulation process. Patient information was anonymized for further analysis after this. Our study was approved by the Medical Ethics Committee of the Erasmus University Medical Center Rotterdam (MEC-2021-0850). None of the patients refused research participation.

### 2.2 Blood collection

Citrated blood (0.129 M sodium citrate) was collected and first centrifuged at 2500 *× g* for 15 minutes, followed by a second centrifugation step at 20,000 *× g* for 10 minutes to obtain platelet-poor plasma, which was frozen in aliquots at −80 °C for further use in the study.

### 2.3 Thrombin generation and turbidity assay

Turbidity and thrombin generation (TG) were measured simultaneously using a VICTOR Nivo plate reader (PerkinElmer). In a 96-well plate, plasma was mixed with 5 pM tissue factor and 4 μM phospholipid mix (platelet poor plasma [PPP] Reagent, Thrombinoscope B.V.). To activate clotting, calcium and thrombin fluorescent substrate (FluCa, Thrombinoscope B.V.) were injected into the well. Samples were quickly mixed, and measurements were started immediately. The fluorescent signal was measured every 30 seconds at an excitation wavelength of 355 nm and a detection emission wavelength of 460 nm for 1 hour. Turbidity was recorded simultaneously at a wavelength of 450 nm.

### 2.4 Laboratory measurements

All assays (Clauss, Thrombin Reagent), PT (Thromborel S), D-dimer (INNOVANCE D-dimer assay), and anti-Xa (HemosIL Liquid Heparin, RT, batroxobin; all reagents were obtained from Siemens Healthcare Diagnostics) were performed on a fully automated coagulation analyzer (Sysmex CS-5100 system, Siemens Healthcare Diagnostics) according to the protocol of the manufacturer. For the PT curves, the change in turbidity was measured over time. The data were extracted as clot waveforms, which were automatically generated by the device. The RT curves were extracted from the clot waveform using a curve extraction tool. This tool was purpose-made (implemented in Python) by one of the authors. Each clot waveform is a diagram within a PDF, with the axes of the diagram having fixed ranges (time, 0-60 seconds; transmitted light, 0-4000 mA fibrinogen concentration). The tool extracts the diagram from the PDF and identifies the axes and the curve. It returns a list of turbidity values corresponding to t = 0, 1, …, 60.

### 2.5 TG and turbidity assay analysis

To infer the fibrinogen concentration in plasma samples from TG and turbidity assay waveforms, we ran multiple simulations of a proprietary computational model for each measured TG curve, using a range of fibrinogen concentrations as the input variable. The light attenuation over time was the output of the model. From these simulations, we obtained a relationship between the simulated maximum increase in light attenuation and a series of fibrinogen concentrations as model input. This reference curve was then used to derive the fibrinogen concentration in the plasma sample from the measured maximum turbidity increase in the turbidity assay measurement.

The proprietary computational model is a system of coupled ordinary differential equations describing multiple fibrin polymerization processes. A list of these processes can be found in the Supplementary material 1 and Supplementary Table 1. Together, the mathematical model consists of 144 ordinary differential equations (ODEs) per states, of which 12 describe enzymatic digestions and 132 the protofibril and fiber formation. This set of coupled ODEs was solved numerically in MATLAB (Mathworks) using a standard ODE solver for stiff problems (ODE23tb). The light attenuation resulting from the formation of fibrin fibers in the assay was then calculated as described by Carr et al. [14,15].

### 2.6 PT turbidity curve analysis

To infer the fibrinogen concentration in plasma samples from the PT turbidity curve, a linear regression model and a 3-layer NN model (with an input layer with 4 inputs; a fully connected [or dense] layer with 3 hidden units and tanh as activation function; and a fully connected output layer with 1 output and a linear [ie, no] activation function) were trained and tested in a leave-one-out method on selected features from the PT turbidity curve (and its first and second derivatives). Extra information on the NN model is provided in Supplementary material 2 and Supplementary Figure 2. As input, different features of the PT turbidity curves were extracted. Specifically, minimum transparency, the maximum absolute value of the transparency gradient, the maximum second derivative of transparency with respect to time, and the minimum second derivative of transparency with respect to time were selected for the linear regression model. For the NN model, the same inputs were used and normalized to zero mean and unit SD for training (and scaled by the same constants for inference). This model was then optimized by minimizing the mean squared error between model output and measured fibrinogen concentrations using Adam optimization with Keras default settings: learning rate = 0.001, beta_1 = 0.9, beta_2 = 0.999, and epsilon = 1e-7. The NN model provided optimal results above a predicted fibrinogen concentration of 6 g/L. When the NN model predicted a lower concentration, the prediction of the linear regression model (for the sample measurement) was more accurate. Based on the results, the optimal combination of the linear regression model (<6 g/L fibrinogen) and the NN model (>6 g/L fibrinogen) was chosen.

### 2.7 RT turbidity curve analysis

The same setup as for the linear regression and NN models was used to analyze the estimation of fibrinogen concentration from RT turbidity curves.

### 2.8 Statistical analysis

To compare modeled fibrinogen levels with measured fibrinogen levels, fibrinogen levels from the models were correlated with Clauss fibrinogen levels. For the analysis, samples were subdivided into “standard” samples and “challenging” samples (eg, INR >1.1, increased D-dimer [> 0.5 mg/L], and increased anti-Xa levels > 0.1 U/mL). Samples that failed to give any test results that were required for model input were excluded from the analysis. A Pearson correlation coefficient was calculated for each method. All tests were 2-tailed, and *P* values < .05 were considered statistically significant. All statistical analyses were performed using IBM SPSS Statistics 28. Graphs were made using Graphpad Prism version 9.

## 3 Results

In total, 44 patient samples were included in this feasibility study. Patients presented with a fibrinogen concentration between 1.1 and 16.6 g/L. For 24 of the 44 patients, a prolonged PT (> 13 seconds) was measured. Four patients had an abnormal D-dimer (> 0.5 mg/L). Finally, 14 patient samples had elevated anti-Xa levels ranging from 0.1 to 1.37 U/mL. The patient with a fibrinogen concentration of 16.6 g/L was removed from the data because the mathematical methods were not equipped to predict such extreme fibrinogen levels.

### 3.1 TG and turbidity

**Figure 3:**
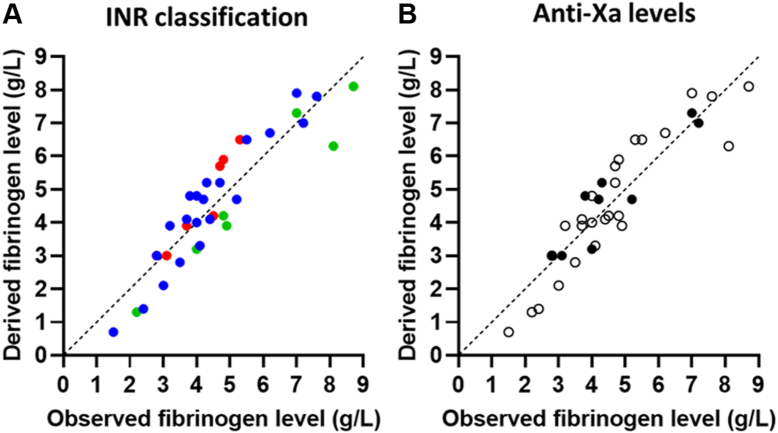
Correlations between the observed fibrinogen levels obtained with the Clauss assay compared with the fibrinogen levels derived from the model of thrombin generation and turbidity-derived data. (A) The correlation between the observed and derived fibrinogen levels, with samples classified as regular (international normalized ratio [INR] 0.9-1.1; blue) and more challenging (INR 1.2-1.5 [green] or INR >1.5 [red]). (B) The same correlation, but with patient samples stratified based on measurable anti-Xa levels (> 0.10 U/mL; black circles) after heparin treatment or no measurable anti-Xa levels (open circles).

In this study, data from multiple clotting assays were tested to derive a fibrinogen concentration mathematically, starting with TG assay measurements that simultaneously measured turbidity levels. Samples with a wide fibrinogen range, between 1.1 and 8.7 g/L, were included. When the inferred fibrinogen levels were compared with those measured by the Clauss assay, a correlation of .917 (*P* < .001) was observed (Figure 3). However, for 8 of the 43 samples, TG and/or turbidity curves were atypical and very low. One of the patients who generated such an atypical and low TG curve had just undergone a liver transplantation. Other patients had high anti-Xa levels > 1.0 U/mL (*n* = 3), were taking DOAC medication (*n* = 1), and/or had an extreme INR of above 7 (*n* = 1). For 2 patients, no clear identification of atypical curves was found in their patient files. When we excluded patients with a higher INR, increased anti-Xa levels, or both, we observed a correlation of .965 (*P* < .001).

### 3.2 PT and turbidity

**Figure 4:**
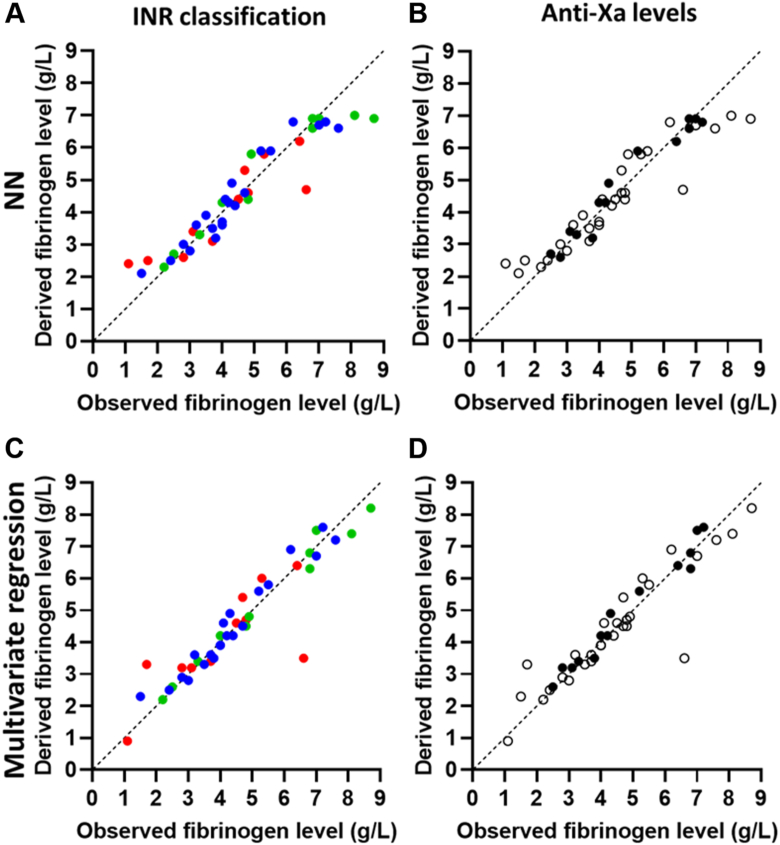
Correlations between the observed fibrinogen levels obtained with the Clauss assay compared with the fibrinogen levels derived from the model of prothrombin time and turbidity-derived data. (A) Neural network (NN) model and (C) multivariate regression model depicting the correlation between the observed and derived fibrinogen levels, with samples classified as regular (international normalized ratio [INR] 0.9-1.1; blue) and more challenging (INR 1.2-1.5 [green] or INR >1.5 [red]). (B) NN model and (D) multivariate regression model depicting the same correlation, but with patient samples stratified based on measurable anti-Xa levels (> 0.10 U/mL; black circles) after heparin treatment or no measurable anti-Xa levels (open circles).

When we evaluated the analysis of the PT clotting curves (across all 43 samples), a correlation coefficient of .945 (*P* < .001) was calculated for the NN model, .944 (*P* < .001) for the multivariate linear regression model, and .965 (*P* < .001) for the combined model. When patient samples with increased INRs and increased anti-Xa levels were excluded, the coefficients slightly changed to .973 (NN; *P* < .001), .984 (multivariate regression; *P* < .001), and .979 (combined; *P* < .001). The correlations between the observed and derived fibrinogen levels for each method are depicted in Figure 4.

### 3.3 RT and turbidity

**Figure 5:**
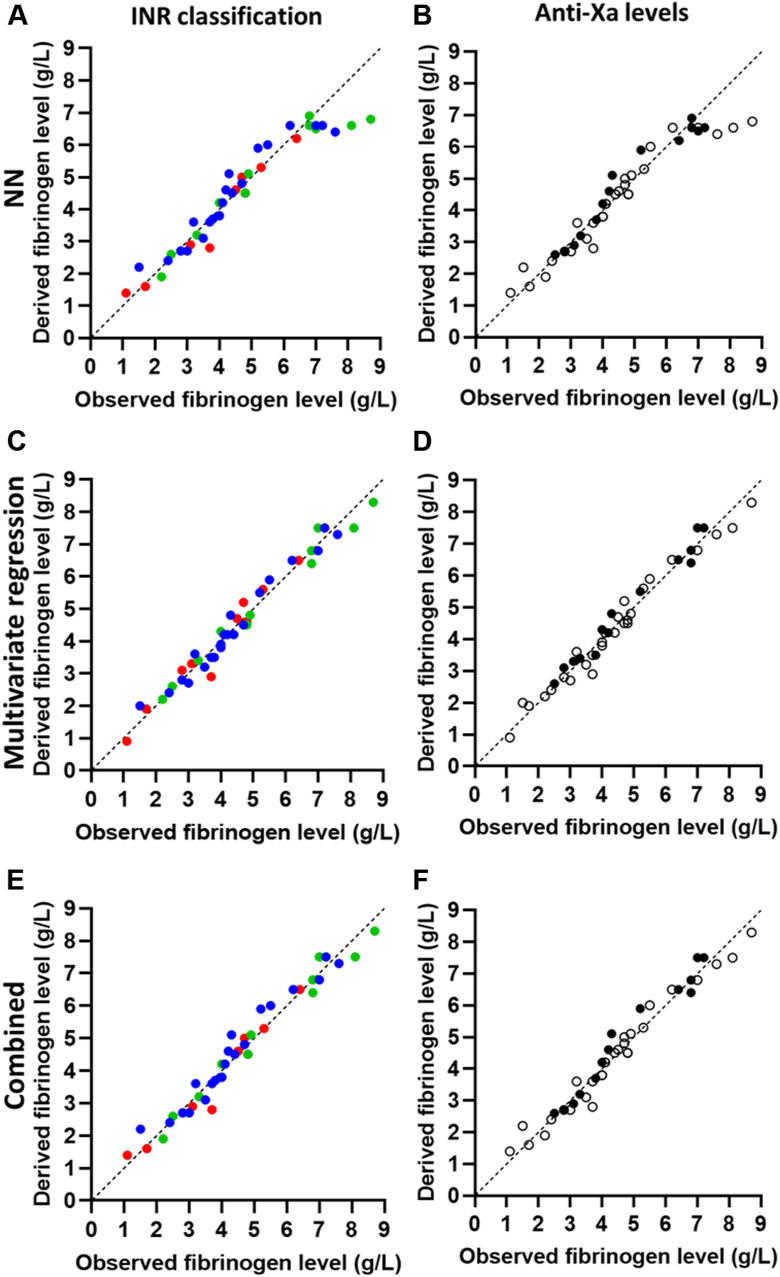
Correlations between the observed fibrinogen levels obtained with the Clauss assay compared with the fibrinogen levels derived from the model of Reptilase time and turbidity-derived data. (A) Neural network (NN) model, (C) multivariate regression model, and (D) combined model depicting the correlation between the observed and derived fibrinogen levels, with samples classified as regular (international normalized ratio [INR] 0.9-1.1; blue) and more challenging (INR 1.2-1.5 [green] or INR >1.5 [red]). (B) NN model, (D) multivariate regression model, and (F) combined model depicting the same correlation, but with patient samples stratified based on measurable anti-Xa levels (> 0.10 U/mL; black circles) after heparin treatment or no measurable anti-Xa levels (open circles).

We also tested the RT clotting curves with 3 different models for fibrinogen level prediction. A clotting curve was obtained for 42 of 43 samples. When we compared the samples with the fibrinogen range, the correlation coefficient was calculated of 0.960 (*P* < .001) for the NN model, 0.985 (*P* < .001) for the multivariate model, and 0.981 (*P* < .001) for the combined model. When patient samples with increased INRs and anti-Xa levels were excluded, the coefficients changed to 0.970 (NN; *P* < .001), 0.989 (multivariate regression; *P* < .001), and 0.985 (combined; *P* < .001). Figure 5 depicts the correlations between the measured and modeled fibrinogen levels for all 3 models.

## 4 Discussion

In this study, we verified the feasibility to determine fibrinogen concentrations by mathematical analysis of TG, PT, and RT waveforms in real-world samples. We compared these inferred concentrations with measured fibrinogen levels performed with the Clauss assay.

Our first method combined input of the TG and turbidity assays. For most of the tested samples, an accurate fibrinogen level was obtained with our model. For 8 samples, an atypical, low TG curve was generated. The lack of TG could be mainly attributed to the presence of anticoagulants in the plasma, including dabigatran, vitamin K antagonists, or heparin. There seems to be a trend in which lower fibrinogen levels are underestimated. Because the number of samples with low fibrinogen levels (< 2.5 g/L) was limited, more samples need to be tested in future studies. As the model uses machine-based learning, measuring samples with lower fibrinogen levels and, similarly for samples with high fibrinogen levels will most likely contribute to a more accurate model.

Our second method combined the input of the PT and turbidity assays. When the NN model was used, the correlation between the modeled and measured fibrinogen levels appeared to have an S-curve. Consequently, higher fibrinogen levels were underestimated, and lower fibrinogen levels were overestimated. This S-curve shape is also a well-known problem of the commercially available PT-based fibrinogen assay as well.[7,16,17]. Therefore, guidelines on the use of fibrinogen assays recommend not to use the PT assay for fibrinogen measurements [6]. However, this problem was solved when the multivariate regression method was applied. Then, only 1 sample, a patient with an INR of 7.1, was underestimated. This model was also able to accurately predict higher fibrinogen levels up to 9.0 g/L, in contrast to the Clauss assay, which has an upper limit of 5.0 g/L and requires dilution of the sample above this concentration. One of the limitations of this study was that we were not able to test the performance of the model with plasma samples of patients with dysfibrinogenemia. Previous research highlighted that the PT-derived fibrinogen assay is not suitable for patients with dysfibrinogenemia [5]. Further research should clarify whether this mathematical model is also applicable to these patients. A second limitation of the study is that data on the ethnicity of the patients were unknown. Therefore, this patient group is only a representation of the patient cohort treated at the Erasmus Medical Center in Rotterdam, the Netherlands.

Our third method combined the input of the RT and turbidity assays. Correct fibrinogen concentrations could be determined for all patient samples except for a patient with an extreme prolonged INR outside of the therapeutic window. The multivariate regression and combined analysis methods showed the highest accuracy, with correlation coefficients of 0.989 (multivariate regression) and 0.985 (combined) for a large range of fibrinogen levels. Among the tested methods in this paper, the combined method showed the highest overall correlation between the inferred fibrinogen values and the measured Clauss fibrinogen values.

Although the results from this study are encouraging, some practical aspects need further consideration and investigation. For instance, the TG, turbidity, PT, and RT curves were all measured in plasma samples, while POC assays typically use whole blood. This may be side-stepped using blood cell filters or solved mathematically. As we have shown, multiple mathematical approaches gave good correlations between inferred and measured fibrinogen concentrations. Hence, we have multiple options to successfully transition the assay from plasma to whole blood. Recently, an improved method of the whole blood TG has been introduced [18]. Further testing and validation will be needed for the developed whole-blood assay as well, using a variety of challenging samples, such as samples with low fibrinogen levels (< 2.0 g/L) and samples from patients with dysfibrinogenemia.

Within this feasibility study, we took the first step toward the development of a new fibrinogen assay. In conclusion, our mathematical models proved to accurately predict a wide range of fibrinogen levels in real-world patient samples with the input of TG, PT, and RT data. The robustness of the PT and RT assays was greater than the TG assay in the presence of anticoagulant reagents. Today, while there are multiple fibrinogen assays available on the market, the options for fast, POC fibrinogen systems are still limited. In this feasibility study, we took the first step toward the development of such a new POC fibrinogen assay.
