# Supplementary material for: Feasibility to infer accurate fibrinogen concentrations from thrombin generation, prothrombin time, or Reptilase time waveforms
Source: Res Pract Thromb Haemost. 2025 Nov 19;9(8):103269. doi: 10.1016/j.rpth.2025.103269 (PMC12753218; doi:10.1016/j.rpth.2025.103269)
Supplement: Supplementary Material 1 [file mmc1.docx]

**Supplemental material**

**Feasibility to infer accurate fibrinogen concentrations from thrombin generation, prothrombin-time or reptilase-time waveforms.**


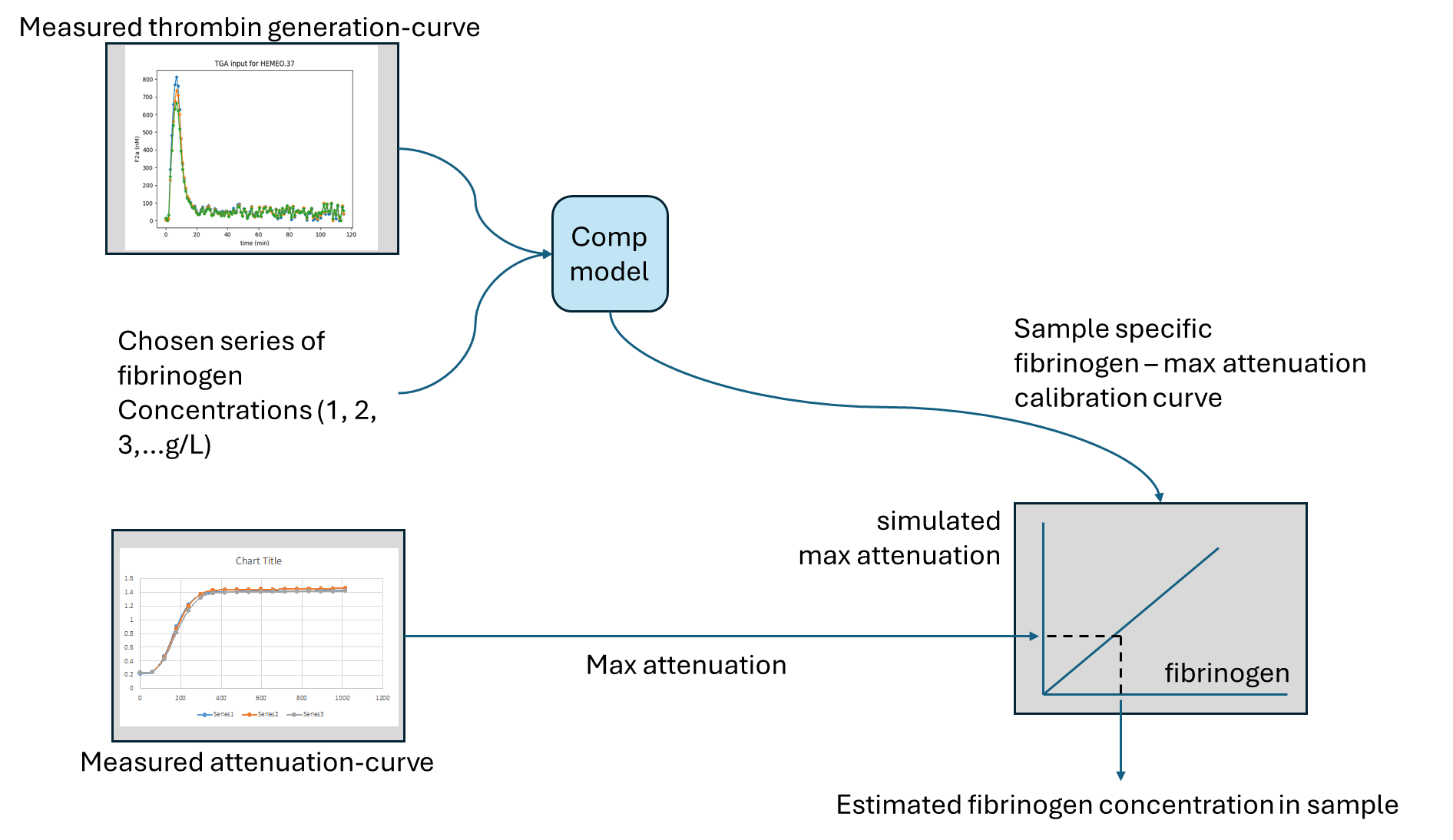


Supplementary figure 1. Analytic workflow from a patient sample to infer the fibrinogen concentration based on data input from the measured thrombin generation and attenuation curve.

Supplementary information on the equations used within the model to describe fibrin polymerization

To infer the fibrinogen concentration in plasma samples from the thrombin generation and turbidity assay waveforms, the thrombin concentration present in the assay over time was used as the input to simulate fibrin fiber formation using a proprietary computational model of coupled ordinary differential equations describing the following fibrin polymerization processes:

- The cleavage of fibrinopeptide A from the fibrinogen molecule by a single binding thrombin molecule.
- The cleavage of fibrinopeptide B from the fibrinogen molecule by a single binding thrombin molecule.
- The cleavage of fibrinopeptide A from the partially activated desBB fibrin monomer by a single binding thrombin molecule.
- The cleavage of fibrinopeptide B from the ~~partially activated~~ desAA fibrin monomer by a single binding thrombin molecule.
- The cleavage of fibrinopeptide A from the fibrinogen molecule by a second binding thrombin molecule.
- The cleavage of fibrinopeptide B from the fibrinogen molecule by a second binding thrombin molecule.
- The initiation of the formation and growth of protofibrils
- The initiation of the formation and growth of fibrils

Note: Additional information on the incorporated reactions in the fibrin polymerization model can be found in the patent (*[https://patentimages.storage.googleapis.com/45/09/6c/e7b3b7a8928bfb/WO2016097234A1.pdf](https://eur01.safelinks.protection.outlook.com/?url=https%3A%2F%2Fpatentimages.storage.googleapis.com%2F45%2F09%2F6c%2Fe7b3b7a8928bfb%2FWO2016097234A1.pdf&data=05%7C02%7Cr.arisz%40erasmusmc.nl%7C0a69df5297bc4362e31908dd86f260e2%7C526638ba6af34b0fa532a1a511f4ac80%7C0%7C0%7C638815098135398557%7CUnknown%7CTWFpbGZsb3d8eyJFbXB0eU1hcGkiOnRydWUsIlYiOiIwLjAuMDAwMCIsIlAiOiJXaW4zMiIsIkFOIjoiTWFpbCIsIldUIjoyfQ%3D%3D%7C0%7C%7C%7C&sdata=do14IWv0AENCZ72RuwAZAvY05hZ3BXK%2FZy9PbsHdhy4%3D&reserved=0)*)

Supplementary table 1. Reactions incorporated in the fibrin polymerization model.


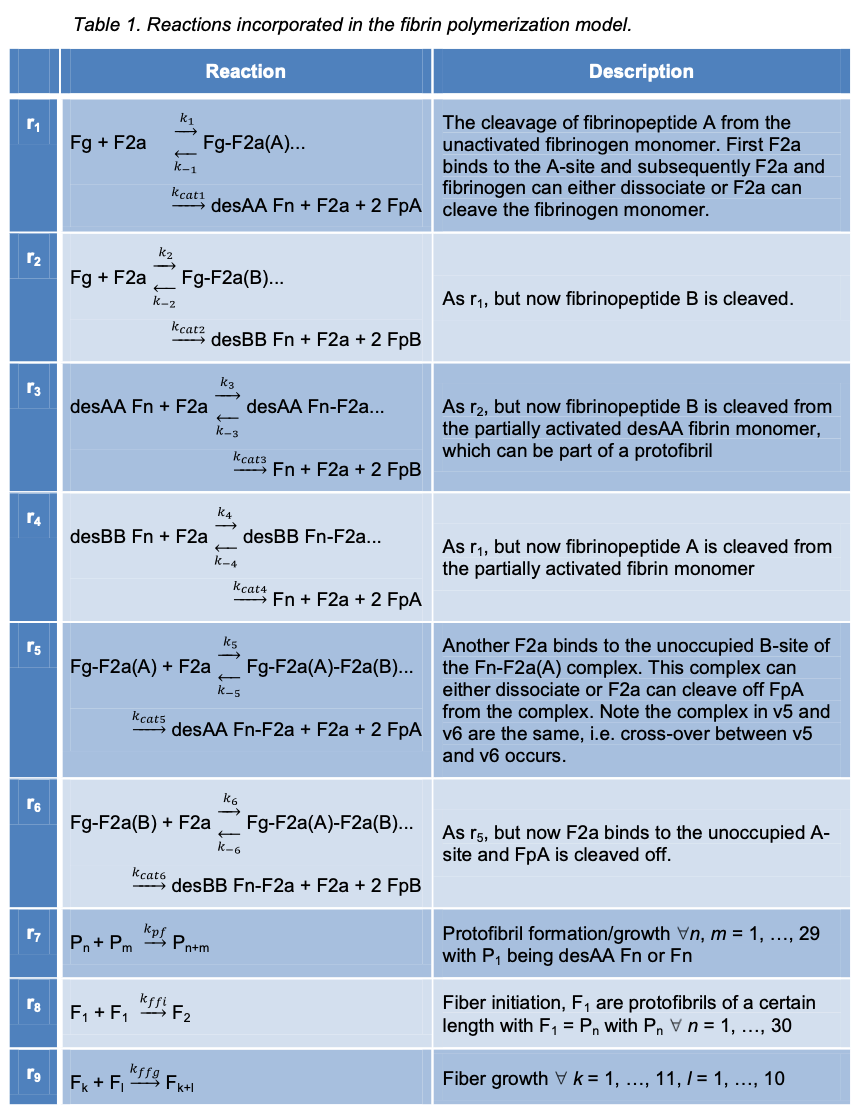


Supplementary information on the Machine Learning Analysis

A 3-layer neural network model was trained for each data subset in a leave-one-out method. This network has an input layer of 4 inputs, a fully connected (also called dense) layer with 3 hidden units and tanh as activation function, and a fully connected output layer of 1 output with a linear (i.e., no) activation function.

The network inputs were normalized to have zero mean and unit standard deviation for training (and scaled by the same constants for inference). This model was then optimized by minimizing the mean squared error between model output and measured fibrinogen concentrations, using Adam optimization with (Keras default) settings learning rate = 0.001, beta_1 = 0.9, beta_2=0.999 and epsilon=1e-7.

Each network (one network for each of the leave-1-out data sub-sets) was trained for 500 epochs (training cycles) with a mini-batch size of 5 samples. (Mini batch: for each epoch, all train samples are shuffled into a random order and gradient descent optimization steps are taken on mini-batches of 5 samples at a time, until all samples in the training set have been used).

Each training-validation set (i.e. all data except the single leave-1-out patient on which the trained model is tested) was randomly divided into 90% train data and 10% validation data. The graph below shows a representative example of the reduction of both train and validation error, which shows that 500 epochs is sufficient training time for model training to converge. Both train and validation error strictly go down during the full training run, indicating the absence of over-fitting (extreme fitting of the train data at the cost of generalization on the validation data).

Supplementary figure 2. The mean squared error of the model was effectively reduced when the model was trained with a total of 500 epochs.
